# Lentiviral CRISPR/Cas9 nickase vector mediated BIRC5 editing inhibits epithelial to mesenchymal transition in ovarian cancer cells

**DOI:** 10.18632/oncotarget.21863

**Published:** 2017-10-17

**Authors:** Guannan Zhao, Qinghui Wang, Qingqing Gu, Wenan Qiang, Jian-Jun Wei, Peixin Dong, Hidemichi Watari, Wei Li, Junming Yue

**Affiliations:** ^1^ Department of Pathology and Laboratory Medicine, University of Tennessee Health Science Center, Memphis, USA; ^2^ Center for Cancer Research, University of Tennessee Health Science Center, Memphis, USA; ^3^ Department of Pharmaceutical Sciences, University of Tennessee Health Science Center, Memphis, USA; ^4^ Department of Pathology, Department of Obstetrics and Gynecology, Northwestern University School of Medicine, Chicago, USA; ^5^ Center for Developmental Therapeutics, Chemistry of Life Processes Institute, Northwestern University, Evanston, USA; ^6^ Department of Women’s Health Educational System, Hokkaido University School of Medicine, Hokkaido University, Sapporo, Japan; ^7^ Department of Gynecology, Hokkaido University School of Medicine, Hokkaido University, Sapporo, Japan

**Keywords:** BIRC5 (survivin), CRISPR/Cas9 nickase, lentiviral vector, ovarian cancer, epithelial to mesenchymal transition

## Abstract

BIRC5 encodes the protein survivin, a member of the inhibitor of apoptosis family. Survivin is highly expressed in a variety of cancers but has very low expression in the corresponding normal tissues, and its expression is often associated with tumor metastasis and chemoresistance. We report that survivin was highly expressed in ovarian cancer and strongly correlated with patient overall poor survival. For the first time, we provide experimental evidence that survivin is involved in epithelial to mesenchymal transition (EMT) in ovarian cancer cells. Lentiviral CRISPR/Cas9 nickase vector mediated BIRC5 gene editing led to the inhibition of EMT by upregulating epithelial cell marker, cytokeratin 7 and downregulating mesenchymal markers: snail2, β-catenin, and vimentin in both ovarian cancer SKOV3 and OVCAR3 cells. Consistent with this molecular approach, pharmacological treatment of ovarian cancer cells using a small molecule survivin inhibitor, YM155 also inhibited EMT in these ovarian cancer cell lines. Overexpression of BIRC5 promoted EMT in SKOV3 cells. Using molecular or pharmacological approaches, we found that cell proliferation, migration, and invasion were significantly inhibited following BIRC5 disruption in both cell lines. Inhibition of BIRC5 expression also sensitized cell responses to paclitaxel treatment. Moreover, loss of BIRC5 expression attenuated TGFβ signaling in both SKOV3 and OVCAR3 cells. Collectively, our studies demonstrated that disruption of BIRC5 expression inhibited EMT by attenuating the TGFβ pathway in ovarian cancer cells.

## INTRODUCTION

Ovarian cancer is therapeutically challenging and has the highest mortality rate among gynecological malignancies. It has been estimated that there will be 22,440 new ovarian cancer cases and 14,080 deaths in 2017 in the United States [1]. Ovarian cancer is rarely detected early, and the majority of patients are already at advanced stages at the time of diagnosis. Moreover, ovarian cancers primarily metastasize through peritoneal dissemination from the primary tumor site to other distant organs including the omentum, colon, and liver. This metastatic process is often accompanied by ascitic fluid accumulation, which is associated with tumor metastasis and chemoresistance, further contributing to a poor survival rate in patients [2, 3]. The current clinical therapy is to perform debulking surgery followed by chemotherapy [4]. However, ovarian cancers are frequently recurrent, and become chemoresistant once recurrence occurs. The current therapeutic approaches are limited due to a lack of understanding the molecular mechanisms underlying tumor metastasis and chemoresistance.

Epithelial to mesenchymal transition (EMT) is a biological process whereby epithelial cells acquire mesenchymal phenotypes by losing their polarity and cell-cell adhesion and gain migratory or invasive properties [5]. During the EMT process, epithelial markers such as E-cadherin, are downregulated, and mesenchymal markers such as snai2, vimentin, and β-catenin are upregulated [6]. The EMT process is regulated by multiple signaling pathways, including TGFβ, WNT, HMGA2 and NF-kB [6–9]. Extensive studies have indicated that EMT contributes to tumor metastasis and chemoresistance [10–12]. In ovarian cancers, EMT is also associated with peritoneal metastasis and chemotherapy drug resistance and with a poor survival rate in patients [6, 12–15].

BIRC5 (baculoviral IAP repeat containing 5) encodes survivin as the smallest member of the inhibitors of apoptosis family. Survivin is highly expressed in various human cancers, including ovarian cancer, and its expression is very low in fully differentiated normal adult tissues [16]. Survivin expression is associated with tumor metastasis and chemoresistance in a number of cancer types such as melanoma, renal, prostate, and breast [17–20]. In ovarian cancer, survivin has also been shown to contribute to tumor metastasis and chemoresistance [12, 21–24]. However, the molecular mechanism underlying survivin-associated tumor metastasis and chemoresistance is still not well understood.

In this study, we analyzed survivin expression in ovarian cancer patient samples and report that survivin was highly expressed in ovarian cancer and associated with poor survival. Lentiviral CRISPR/Cas9 nickase vector mediated BIRC5 editing led to the inhibition of EMT in ovarian cancer SKOV3 and OVCAR3 cells. Inhibition of BIRC5 expression by using a small molecule inhibitor of survivin, YM155, also suppressed EMT in both ovarian cancer cell lines. By using molecular or pharmacological approaches, intervening BIRC5 expression led to an inhibition of cell proliferation, migration, and invasion and sensitized cell response to chemotherapy drug treatment. In addition, inhibition of BIRC5 expression attenuated the TGFβ pathway in both SKOV3 and OVCAR3 cells. Our results indicated that survivin may contribute to tumor metastasis and chemoresistance by promoting EMT through the TGFβ pathway in ovarian cancer cells.

## RESULTS

### BIRC5 was highly expressed in ovarian cancer and associated with poor survival in patients

**Figure 1:**
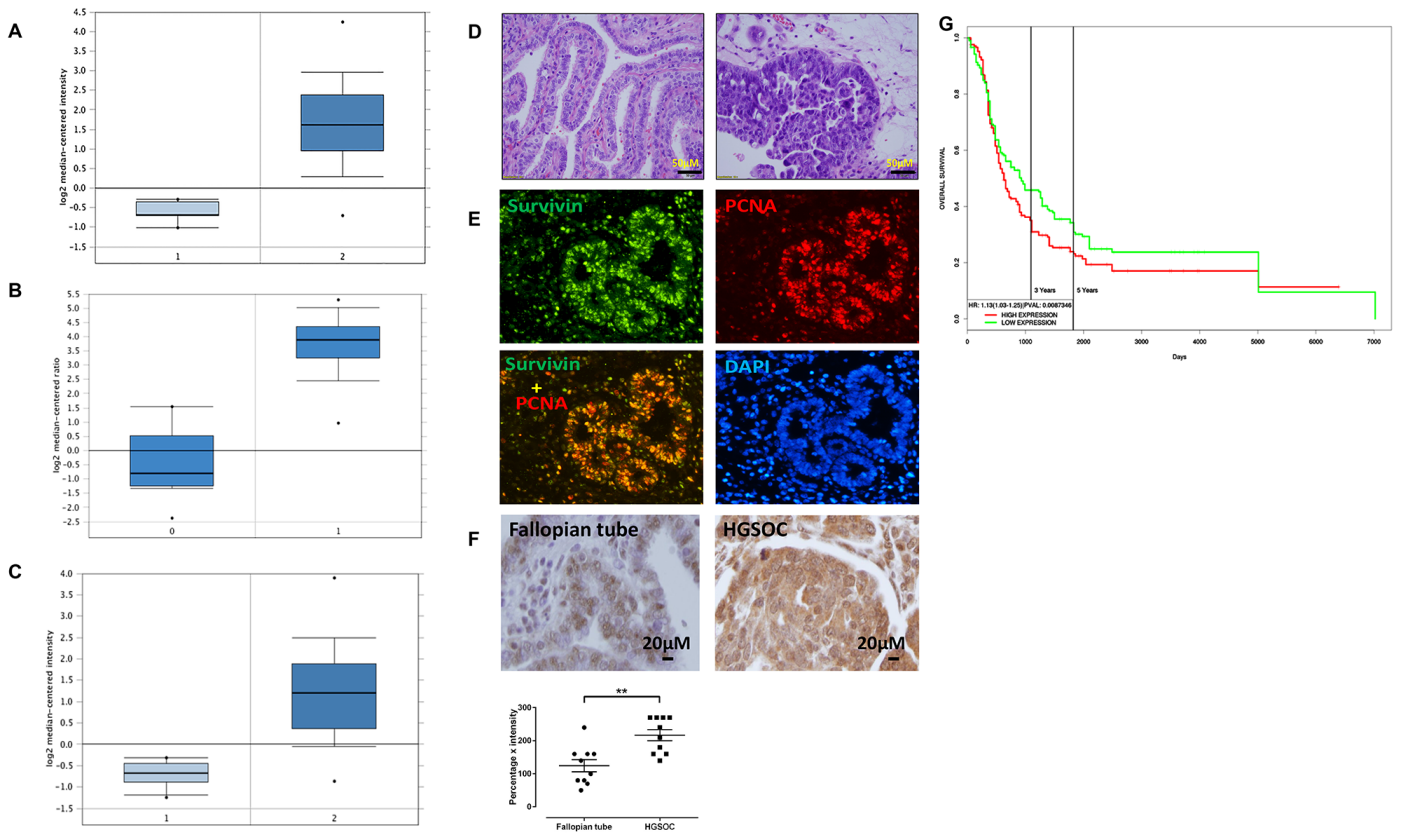
BIRC5 was highly expressed in ovarian serous carcinoma and associated with poor patient survival **(A)** 1 = Normal ovary tissue (N=8); 2 = Ovarian cancer (N=586). **(B)** 1 = Normal ovarian surface epithelium (N=10); 2 = Ovarian cancer (N=185). **(C)** 1 = Normal peritoneum tissue (N=10); 2= Ovarian cancer (N=43). **(D)** H.E. staining of ovarian serous carcinoma in low and high magnification. **(E)** Immunofluorescent staining of survivin and PCNA in sections of ovarian serous carcinoma. **(F)** Immunohistochemical staining of survivin in normal fallopian tubes and high grade serous ovarian carcinoma (n=10, **p<0.01). **(G)** BIRC5 expression and ovarian patient survival in ovarian serous carcinoma (n=207, p=0.0008).

To examine the expression level of BIRC5 in ovarian cancer, we analyzed TCGA datasets that included 586 serous ovarian carcinomas and 8 normal ovary controls [25]. BIRC5 expression was significantly higher by approximately a 5-fold increase in tumors than in controls (*P*=2.20E-10) (Figure 1A). We also analyzed 185 ovarian carcinomas and 10 normal ovarian surface epithelia in the Oncomine database [26], and BIRC5 expression was significantly higher in tumors than in controls with approximately a 4-fold increase (Figure 1B). In addition, we examined BIRC5 expression in ovarian cancer in the Oncomine database including 43 serous carcinomas and 10 peritoneal controls [27]; BIRC5 expression showed approximately a 20-fold increase in tumors compared to controls (*P*=5.68E-8) (Figure 1C). To confirm the findings of BIRC5 expression in ovarian cancer, we performed immunofluorescent staining on tumor sections of high grade serous ovarian carcinoma (Figure 1D) and found strong immunoreactivity for survivin in tumor cell nuclei but absent in the adjacent normal tissues (Figure 1E). We also examined survivin expression by immunohistochemistry and results indicated that survivin expression was significantly higher in HGSOC than in fallopian tubes (Figure 1F). In data set with information of clinical outcome (Gene Expression Omnibus (GSE13876) from 414 ovarian cancer patients), 207 patients were classified into the high expression group and the other 207 were classified into the low expression group by the median. Based on PROGgene program analysis [28], patients with high expression of survivin was significantly associated with poor survival and prognosis (*P* = 0.008, Figure 1G). All these data suggest that BIRC5 is highly expressed in high grade serous ovarian cancer and the level of survivin overexpression is associated with poor prognosis.

### Disruption of BIRC5 expression using lentiviral CRISPR/Cas9 nickase mediated editing resulted in the inhibition of EMT in ovarian cancer cells

**Figure 2:**
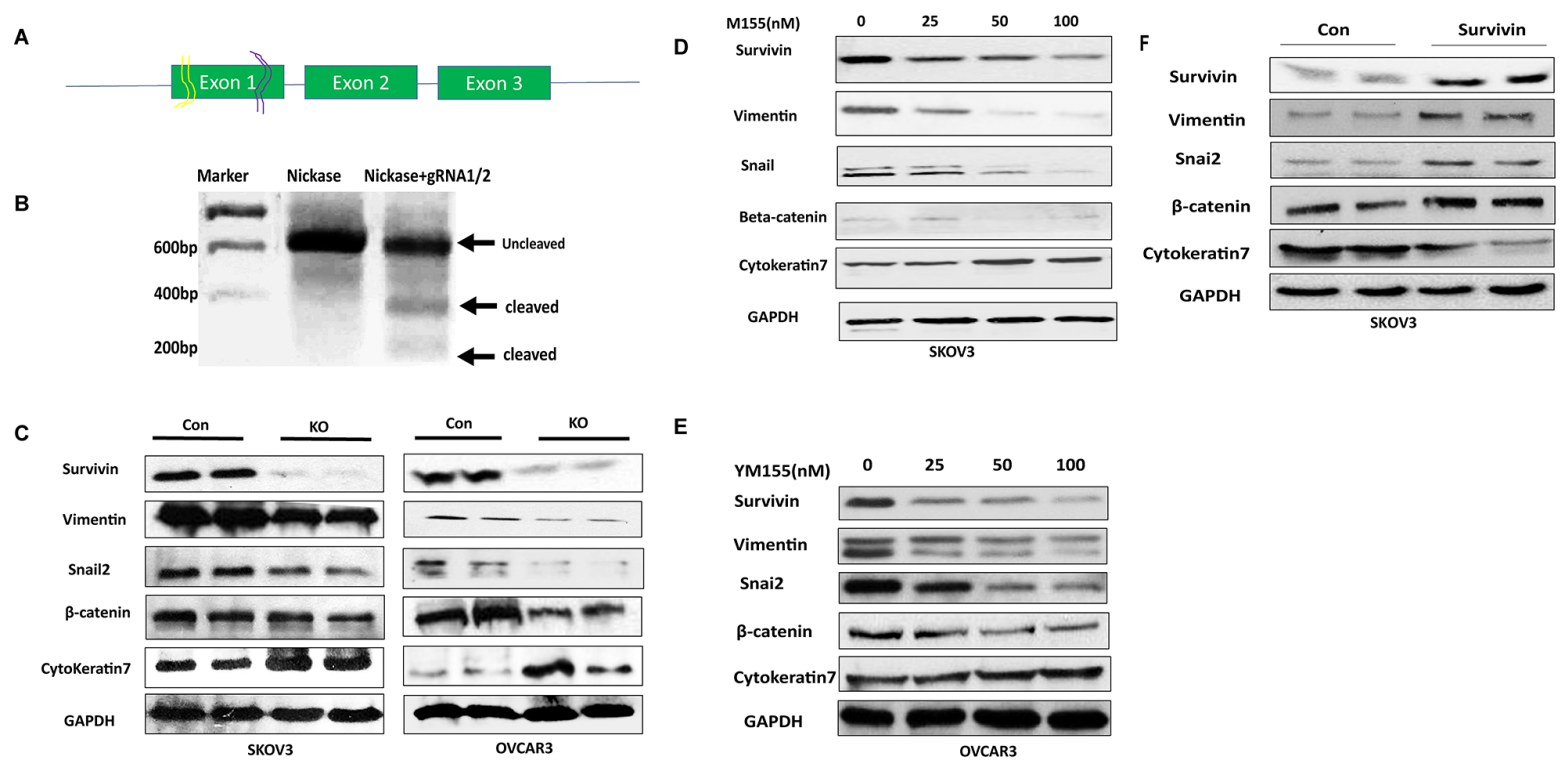
Lentiviral CRISPR/Cas9 nickase-mediated BIRC5 gene editing led to inhibition of EMT in ovarian cancer cells **(A)** Schematic diagram of two gRNAs targeting exon 1 of BIRC5 gene. **(B)** DNA surveyor mutation assay showing two cleaved products presented in BIRC5 knockout, but not in control, cells. **(C)** EMT marker gene expression was examined in BIRC5 knockout and control SKOV3 and OVCAR3 cells by using Western blot. **(D, E)** EMT markers were examined in YM155-treated and control SKOV3 and OVCAR3 cells by Western blot. **(F)** EMT markers were examined by Western blot in BIRC5 expressing and control SKOV3 cells.

To disrupt BIRC5 expression in ovarian cancer cells, we examined endogenous BIRC5 expression in several ovarian cancer cell lines including SKOV3, OVCAR3, Hey and UACC1598 by western blot. Survivin was detected in all of them, and higher BIRC5 expression was found in SKOV3 and UACC1598 than Hey and OVCAR3 (Supplementary Figure 1A). SKOV3 and OVCAR3 cell lines were selected for our studies [29]. We constructed lentiviral CRISPR/Cas9 nickase by using two gRNAs targeting a region of exon 1 (Figure 2A) and then transduced both SKOV3 and OVCAR3 cells. The lentiviral CRISPR/Cas9 nickase vector-mediated mutations in SKOV3 cells were confirmed by using a DNA surveyor assay that the cleaved products were visible in cells transduced with BIRC5 gRNA vector but not in the control vector, indicating that BIRC5 mutation in exon 1 was successfully introduced by this approach (Figure 2B). Next, using Western blot, we examined whether the disruption of the BIRC5 gene resulted in alteration of the survivin protein and EMT-associated markers in both ovarian cancer cells. Survivin was remarkably depleted in both SKOV3 and OVCAR3 cells transduced with lentiviral BIRC5 gRNA vector (knockout) compared to control cells, and EMT markers were also altered by an upregulation of epithelial cell marker, cytokeratin-7 and downregulation of mesenchymal marker: vimentin, snai2 and β-catenin compared to control cells (Figure 2C). To examine the EMT phenotype in ovarian cancer cells, we treated SKOV3 cells using 10 ng/ml of TGFβ for 48 h and cell morphology was imaged. These images clearly showed a fibroblast-like mesenchymal morphology in TGFβ induced control cells, but not in the survivin knockout cells, indicating that loss of survivin inhibited TGFβ induced EMT in SKOV3 cells (Supplementary Figure 1B). We further examined EMT marker gene expression by treating both SKOV3 and OVCAR3 cells with different doses of YM155, a small molecule inhibitor of survivin. Following dose-dependent inhibition of survivin, the epithelial cell marker, cytokeratin-7 was upregulated and mesenchymal markers: vimentin, snai2, and β-catenin were downregulated in both SKOV3 and OVCAR3 cells (Figure 2D, 2E). Disruption of BIRC5 with CRISPR/Cas9 nickase or inhibition of BIRC5 with a small inhibitor resulted in the inhibition of EMT in both SKOV3 and OVCAR3 cells. After BIRC5 was overexpressed using lentiviral overexpression vector in SKOV3 cells, survivin and EMT markers were examined by Western blot. We observed an upregulation of vimentin, snail2 and β-catenin and a downregulation of cytokeratin-7 following BIRC5 overexpression, indicating that survivin expression promoted EMT in ovarian cancer SKOV3 cells (Figure 2F).

### Disruption of BIRC5 expression led to the inhibition of cell proliferation and survival in ovarian cancer cells

**Figure 3:**
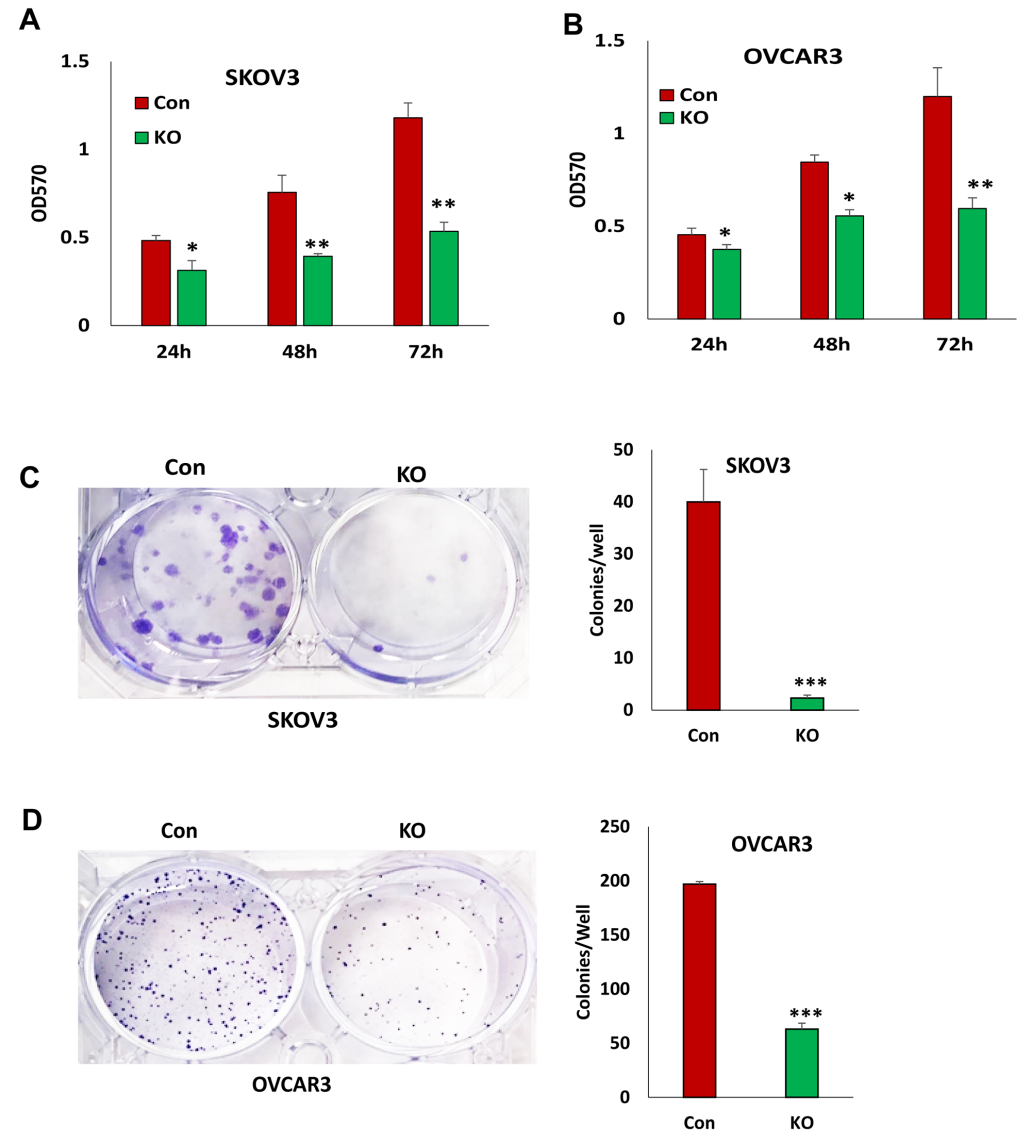
Lentiviral CRISPR/Cas9 nickase-mediated BIRC5 gene editing reduced cell proliferation in ovarian cancer cells **(A, B)** Cell proliferation in BIRC5 knockout and control SKOV3(A) and OVCAR3 (B) cells at different time points was determined by MTT assay (^*^P<0.05;^**^P<0.01). **(C, D)** Cell survival in BIRC5 knockout SKOV3 (C) and OVCAR3 (D) cells was determined by colony formation assay (^***^P<0.001).

**Figure 4:**
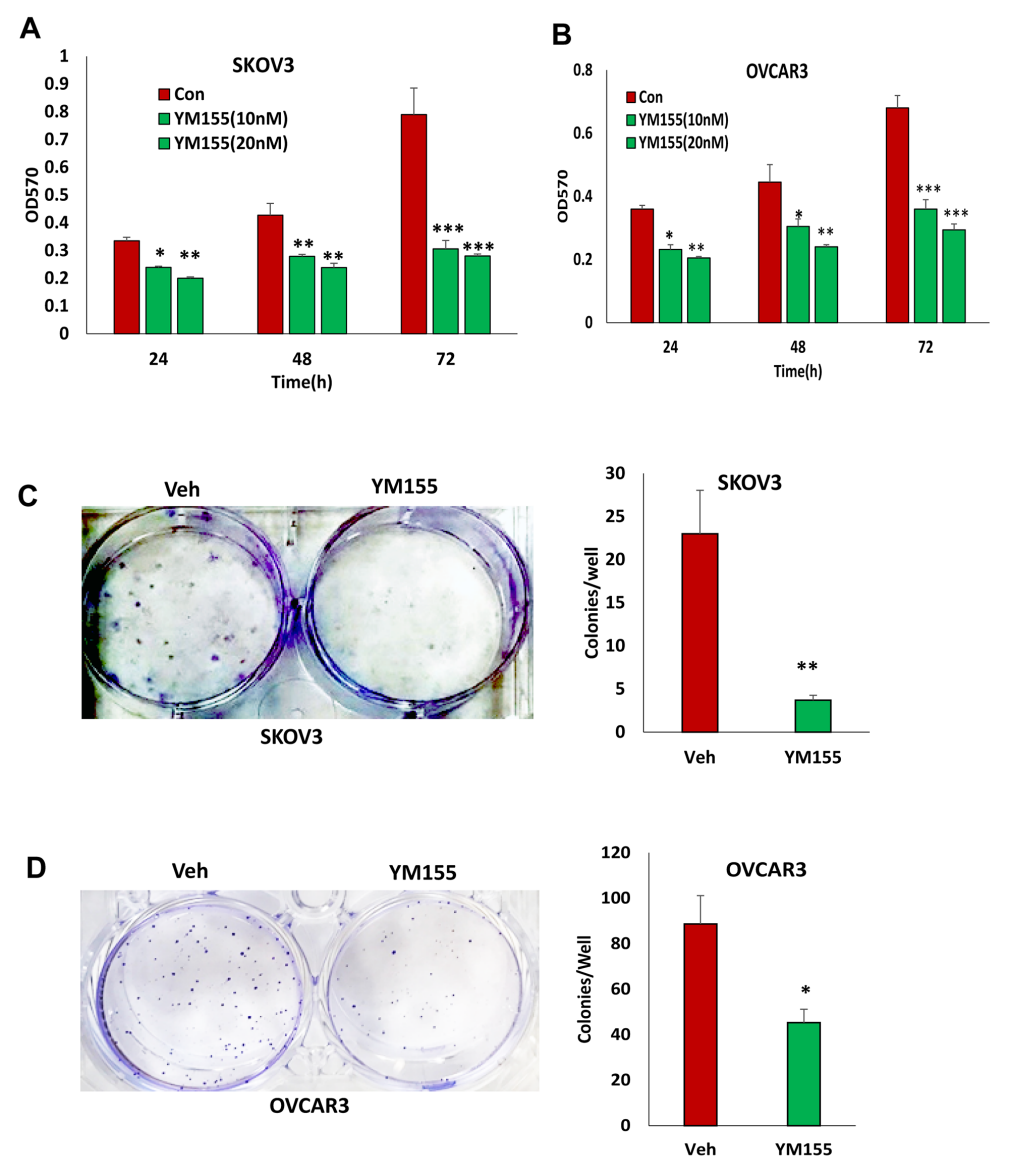
Inhibition of BIRC5 expression using YM155 reduced cell proliferation in ovarian cancer cells **(A, B)** Cell proliferation in wild type ovarian cancer SKOV3 (A) and OVCAR3 (B) cells was determined following treatment using 10 and 20 nM YM155 at different time points by MTT assay (^*^P<0.05;^**^P<0.01;^***^P<0.001). **(C, D)** Cell survival was determined using a colony formation assay following treatment with 5 nM YM155 (^*^P<0.05;^**^P<0.01;^***^P<0.001).

To determine the functional outcome of losing BIRC5 expression in ovarian cancer cells, we examined cell proliferation in SKOV3 and OVCAR3 cells with and without BIRC5 expression. Disruption of BIRC5 by CRISPR/Cas9 nickase significantly reduced cell proliferation compared to controls at all three time points (24, 48, and 72 h) in both SKOV3 and OVCAR3 cells (Figure 3A, 3B). We examined cell survival by assaying cell colony formation; loss of BIRC5 expression led to significant inhibition in both SKOV3 (Figure 3C) and OVCAR3 cells (Figure 3D). In addition, we tested the effect of two different doses of YM155 (0, 10 and 20 nM ) on cell proliferation by treating wild type cells for 24, 48, and 72 h, and cell proliferation was significantly inhibited in both SKOV3 (Figure 4A) and OVCAR3 (Figure 4B) cells. To examine how YM155 affected cell survival, we treated both SKOV3 and OVCAR3 cells using 5 nM of YM155 and found that it significantly inhibited cell survival based on the colony formation assay (Figure 4C, 4D).

### Disruption of BIRC5 expression led to the inhibition of cell migration and invasion in ovarian cancer cells

**Figure 5:**
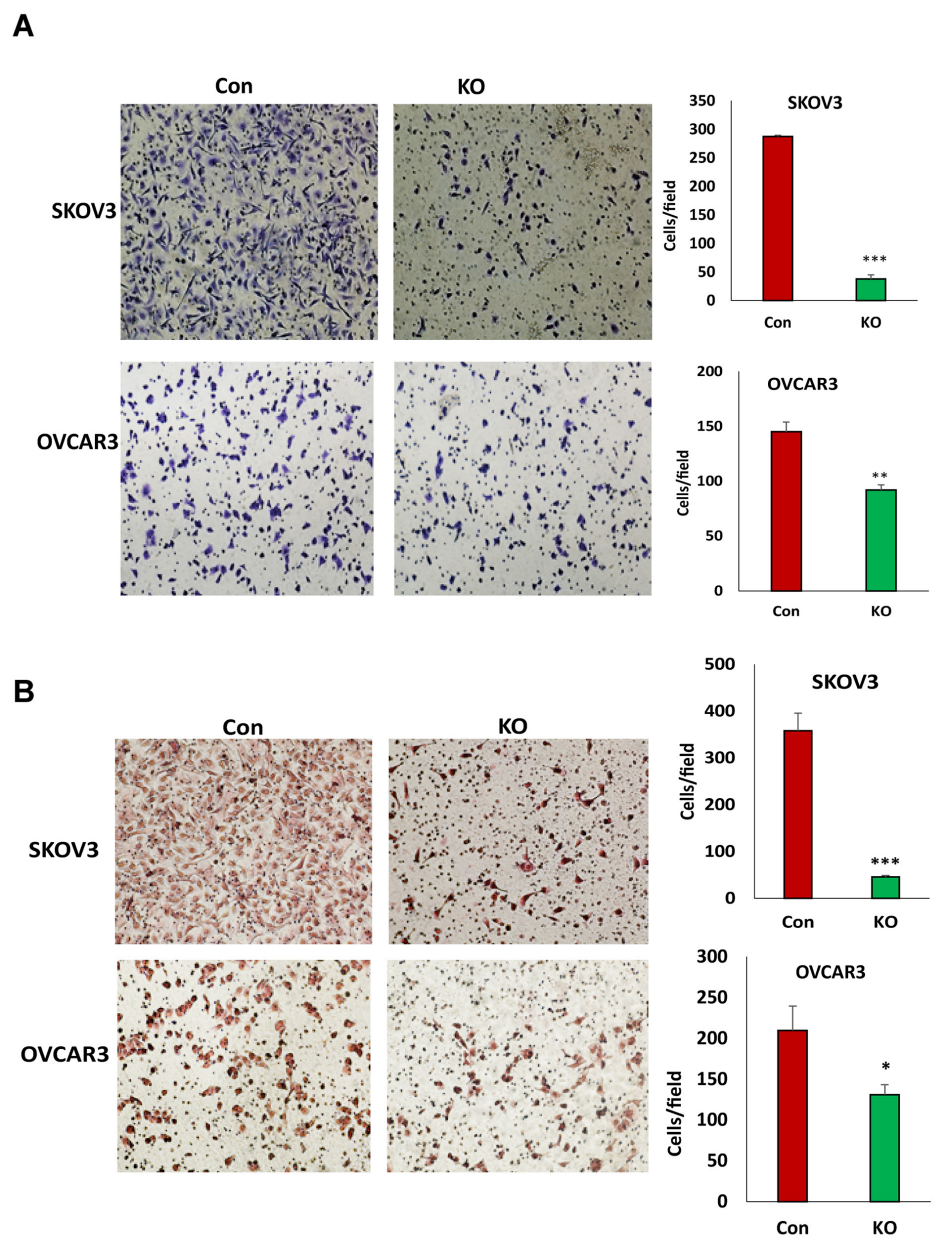
Lentiviral CRISPR/Cas9 nickase mediated BIRC5 gene editing reduced cell migration and invasion in ovarian cancer cells **(A)** Cell migration in BIRC5 knockout and control SKOV3 or OVCAR3 cells was examined using transwell plates, and migrated cells were stained with crystal blue and counted (^**^P<0.01;^***^P<0.001). **(B)** Cell invasion in both BIRC5 knockout and control SKOV3 or OVCAR3 cells was examined using Matrigel-coated plates, and invaded cells were stained with H.E. and counted (^*^P<0.05;^***^P<0.001).

**Figure 6:**
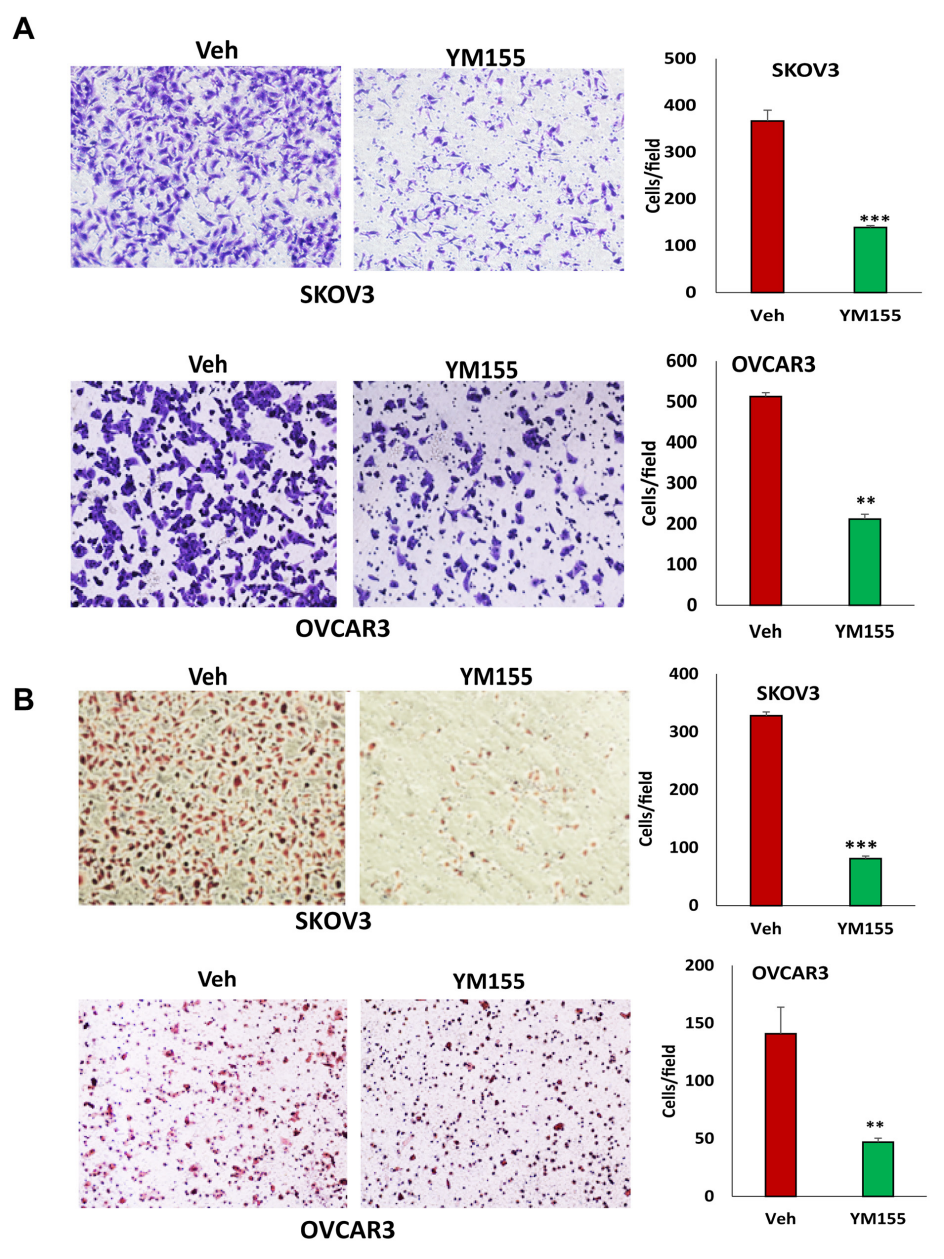
Inhibition of BIRC5 expression using YM155 reduced cell migration and invasion in ovarian cancer cells **(A)** Cell migration in 5 nM YM155- and vehicle-treated SKOV3 and OVCAR3 cells was examined using transwell plates, and migrated cells were stained with crystal blue and counted (^**^P<0.01;^***^P<0.001). **(B)** SKOV3 and OVCAR3 cells were treated with 5 nM YM155 for 4 h, and invasion was examined using Matrigel-coated plates, and invaded cells were stained with H.E. and counted (^*^P<0.05;^***^P<0.001).

The loss of BIRC5 expression inhibited EMT in ovarian cancer cells and suggested that BIRC5 may affect cell motility and invasion. Using transwell plates, we examined cell migration in BIRC5 knockout and control cells and found that cell migration was significantly reduced in both the BIRC5 knockout, SKOV3 and OVCAR3 cells (Figure 5A). Using Matrigel-coated transwells, we assessed cell invasion, which was also significantly reduced in both SKOV3 and OVCAR3 cells compared to the controls (Figure 5B). To test whether survivin inhibitor has a similar effect on cell migration and invasion, we treated both cell lines with 20 nM YM155 for 4 h, and cell migration and invasion were determined with the same methods. Our results showed that inhibition of survivin with YM155 significantly reduced migration and invasion in both cell lines (Figure 6A, 6B).

### Disruption of BIRC5 expression sensitized cell responses to chemotherapy drug treatment

**Figure 7:**
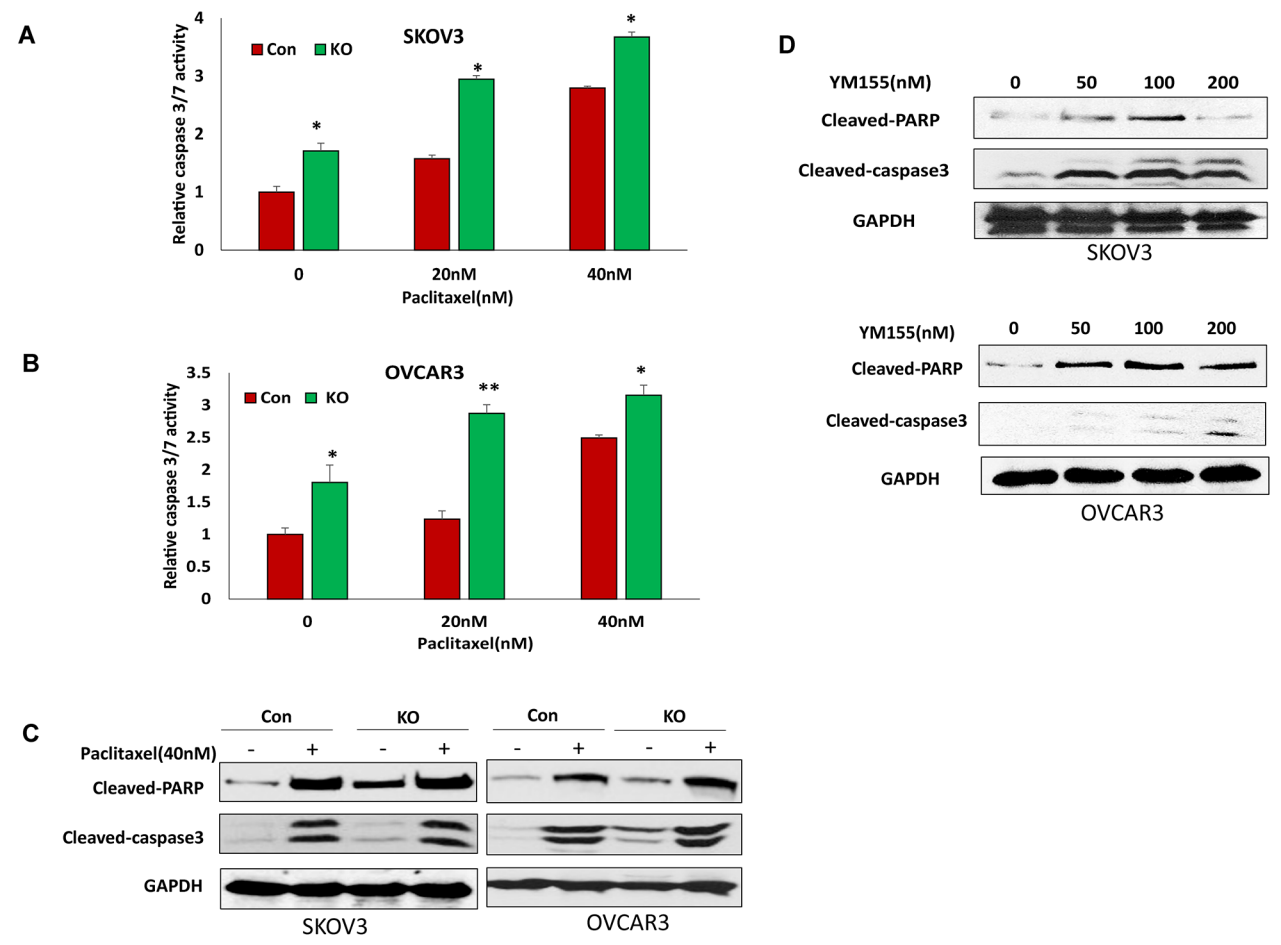
Lentiviral CRISPR/Cas9 nickase-mediated BIRC5 gene editing sensitized cell responses to chemotherapy drug treatment **(A, B)** Cell apoptosis in BIRC5 knockout and control SKOV3 (A) and OVCAR3 (B) cells following 20 and 40 nM paclitaxel treatment for 24 h was determined by measuring caspase3/7 activity (^*^P<0.05;^**^P<0.01). **(C)** Cell apoptosis in BIRC5 knockout and control SKOV3 and OVCAR3 cells following 40 nM paclitaxel treatment for 24 h was examined by determining cleaved PARP and caspase3 using Western blot. **(D)** SKOV3 and OVCAR3 cell apoptosis was examined following treatment using different doses of YM155 by Western blot.

BIRC5 expression in ovarian cancer cells may contribute to chemoresistance. To test whether a loss of BIRC5 expression sensitized the cell response to chemotherapy, we treated BIRC5 knockout and control SKOV3 and OVCAR3 cells with different doses of paclitaxel. Loss of BIRC5 expression induced cell apoptosis as shown by caspase3/7 activity and sensitized cell response to paclitaxel treatment at 20 and 40 nM doses in both SKOV3 (Figure 7A) and OVCAR3 (Figure 7B) cells. Using Western blot, we also detected cell apoptosis by examining cleaved PARP and cleaved caspase3. Both apoptotic proteins were significantly induced by losing BIRC5 expression with or without paclitaxel (Figure 7C). To test whether an inhibition of BIRC5 expression with YM155 also induced cell apoptosis, both SKOV3 and OVCAR3 cells were treated with different doses from 50 to 200 nM; using Western blot, we detected apoptosis by examining cleaved PARP and caspase3 and found that inhibiting BIRC5 expression using YM155 induced cell apoptosis in both ovarian cancer cells (Figure 7D). Our data indicate that disruption of BIRC5 expression enhanced the efficacy of chemotherapy in ovarian cancer cells.

### Loss of BIRC5 expression attenuated TGFβ signaling pathway in ovarian cancer cells

**Figure 8:**
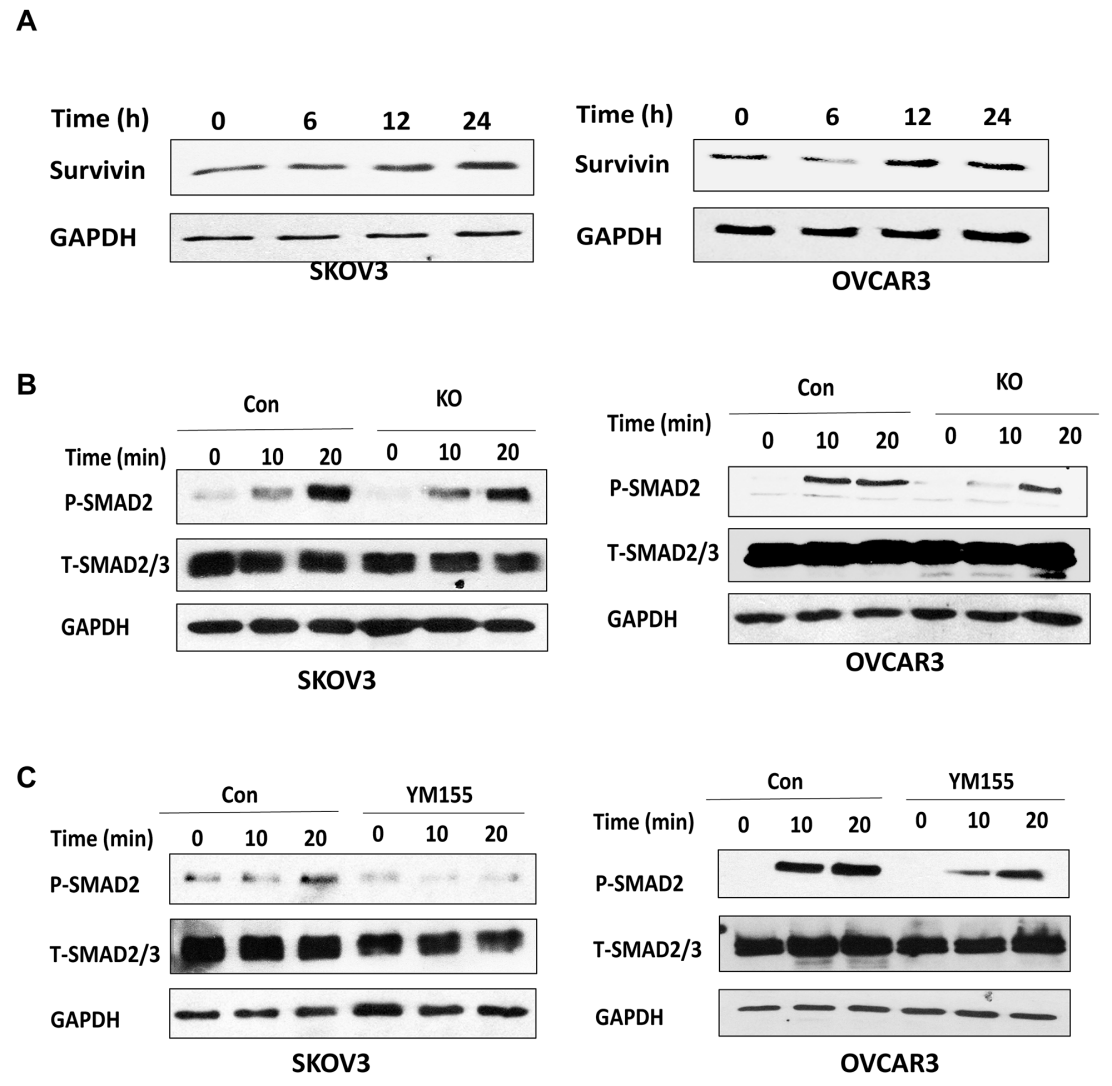
Lentiviral CRISPR/Cas9 nickase-mediated BIRC5 gene editing attenuated the TGFβ pathway in ovarian cancer cells **(A)** TGFβ induced survivin in SKOV3 and OVCAR3 cells at the indicated time points as detected by Western blot. **(B)** The expression of phospho- and total SMAD2 in BIRC5 knockout and control SKOV3 and OVCAR3 cells was detected by Western blot following 10 ng/ml TGFβ treatment at the indicated time points. **(C)** The expression of phospho- and total SMAD2 was detected by Western blot in SKOV3 and OVCAR3 cells following 20 nM YM155 treatment for 24 h and then treated with 10 ng/ml TGFβ at the indicated time points.

As we showed in our previous study, TGFβ promoted EMT in ovarian cancer cells [30]. To understand how BIRC5 regulates EMT in ovarian cancer cells, we tested the correlation between survivin and the TGFβ pathway in ovarian cancer cells. First, we determined BIRC5 expression in both SKOV3 and OVCAR3 cells following TGFβ treatment at different time points (0, 6, 12, 24h ) and found that TGFβ induced survivin expression (Figure 8A). Next, to determine whether BIRC5 expression was involved in the TGFβ pathway we treated BIRC5 knockout and control SKOV3 and OVCAR3 cells at different time points (0, 10 and 20 min) and then examined phospho- and total SMAD2 by using Western blot. Loss of survivin resulted in attenuating the TGFβ signaling pathway in the BIRC5 knockout compared to control cells (Figure 8B). We also determined the TGFβ pathway following 20 nM YM155 treatment and found that inhibition of survivin attenuated phospho-SMAD2 in both SKOV3 and OVCAR3 cells (Figure 8C). Our data indicated that loss of BIRC5 expression attenuated the TGFβ signaling pathway, thereby potentially leading to the inhibition of EMT in ovarian cancer cells.

## DISCUSSION

BIRC5 is an effective drug target for cancer therapy due to its high expression in tumors, but not in normal tissues. BIRC5 was highly expressed in high grade serous ovarian carcinoma compared with ovarian, fallopian tube epithelia, and peritoneal fluid based on a large number of cancer patients from three different databases and current study. Furthermore, BIRC5 overexpression results in EMT alteration and tumor aggressive growth. Thus, targeting BIRC5 by using small molecule inhibitors like YM155 may provide a novel therapeutic approach in ovarian cancer therapy by inhibiting ovarian tumor metastasis and overcoming chemoresistance. BIRC5 expression significantly correlated with overall poor survival in patients based on analyzing data from 514 ovarian carcinomas. The results from our current study are consistent with findings from several previous studies showing that survivin is associated with poor survival and prognosis [31–34]. Although we showed that BIRC5 was highly expressed in high grade serous ovarian carcinoma, the role of BIRC5 expression in other types of ovarian cancer, including mucinous, clear cell, and endometrioid carcinoma was still unknown. Therefore, additional studies are required to determine the correlation of BIRC5 expression with diverse types of ovarian cancer at various disease stages in order to define its potential role in diagnosis, prognosis, and therapy.

Ovarian cancer therapy is limited due to its peritoneal spread behavior and chemoresistance. Understanding the underlying molecular mechanism will help find an effective approach by targeting at this process. In this study, we demonstrated for the first time that BIRC5 expression promoted EMT in ovarian cancer cells, which is consistent with a previous study reported in colorectal cancer [35]. However, it was also reported by another group that survivin inhibited EMT in hepatocellular carcinoma (HCC) [36]. EMT acquisition confers aggressive cellular behavior, which contributes to tumor metastasis and chemoresistance. Therefore, BIRC5 is a promising drug target for ovarian cancer therapy because of its role in EMT and undetectable expression in normal ovarian tissues. We have tested the function of survivin in ovarian cancer cells by disrupting BIRC5 with lentiviral CRISPR/Cas9 nickase mediated gene editing and a small molecule inhibitor, YM155. Disruption of BIRC5 expression by using both approaches resulted in reduced cell proliferation, migration, invasion, and colony formation in both SKOV3 and OVCAR3 cells. However, those functional assays showed that SKOV3 cells were more invasive than OVCAR3 cells, which may also be contributed by higher expression of survivin in SKOV3 than OVCAR3 cells (Supplementary Figure 1). In addition, inhibition of survivin sensitized cell responses to chemotherapy drug treatment, indicating that BIRC5 is a therapeutic target for ovarian cancer therapy. Although no studies have so far reported on survivin and tumor metastasis in orthotopic ovarian cancer animal models, several studies have shown that silencing BIRC5 expression with siRNA, shRNA, or YM155 inhibited cell proliferation and sensitized the cell response to chemotherapy [22, 37–40]. Our data indicate that BIRC5 expression is potentially associated with ovarian tumor metastasis by promoting EMT. We are in the process of testing our hypothesis: inhibiting BIRC5 expression suppresses tumor metastasis in an orthotopic ovarian cancer mouse model by using selective small molecule survivin inhibitors.

Although we showed that the loss of BRIC5 expression led to an inhibition of EMT, the molecular mechanisms by which BIRC5 expression contributes to EMT is still largely unknown. It is well known that TGFβ promotes EMT in a variety of cancers, including ovarian cancer. For the first time, we found that BIRC5 expression was required for activating the TGFβ pathway and inhibiting survivin by using molecular or pharmacological approaches led to attenuating TGFβ signaling in ovarian cancer cells. Our data suggest that survivin promoted EMT by participating in the TGFβ pathway in ovarian cancer, which is consistent with a report in glioblastoma [41]. Previous studies showed that survivin is regulated by TGFβ through the ERK1/2 or PI3/AKT pathways in other cancer types including glioblastoma [41]. ERK1/2 is a positive regulator of EMT in numerous human cancers, including ovarian cancer [42, 43]. However, survivin is downregulated by TGFβ in prostate cancer by downregulating downstream SMAD2/3, thereby inducing cell apoptosis [44], suggesting that TGFβ-regulated survivin expression depends on cellular context. Therefore, it is possible that survivin-mediated EMT may occur through the TGFβ, ERK1/2, and PI3/AKT pathways in ovarian cancer cells. In this study, we revealed that TGFβ activated survivin expression, and that loss of survivin attenuated TGFβ pathway, suggesting a positive feedback loop or crosstalk between survivin and TGFβ pathway, although it is not clear how survivin is involved in TGFβ pathway. In a previous study, XIAP (X-linked inhibitor of apoptosis protein), a member of the inhibitor of apoptosis family of proteins (IAP) was shown to directly interact with TGFβ receptor1 (TGFβR1) through its baculovirus IAP repeat (BIR) domain [45]. Based on our data that showed the loss of survivin attenuated SMAD2, survivin may regulate TGFβ pathway through those components of upstream SMAD2, such as TGFβR1. Therefore, it is possible that survivin as one of member IAP family may interact with TGFβR1 through its BIR domain, thus activate TGFβ pathway in ovarian cancer cells.

In conclusion, our study demonstrated that BIRC5 is highly expressed in ovarian cancer cells and associated with a patient’s poor survival or prognosis. Disrupting BRIC5 expression by using the lentiviral CRISPR/Cas9 nickase vector or small molecule inhibitor, YM155, inhibited EMT and significantly reduced cell growth and invasion and induced cell apoptosis. Loss of BIRIC5 expression attenuated the TGFβ pathway. Therefore, BRIC5 may contribute to EMT by potentially participating in the TGFβ pathway in ovarian cancer cells.

## MATERIALS AND METHODS

### Cell culture

Ovarian cancer cell lines, SKOV3, OVCAR3, Hey and UACC1598 were obtained from ATCC and cultured in Dulbecco’s Modified Eagle Medium (DMEM) supplemented with 10% FBS (Hyclone; Logan, UT), 100 U/ml penicillin, and 100 μg/ml streptomycin (Invitrogen; Carlsbad, CA). HEK293 FT cells were cultured in DMEM supplemented with 10% FBS, 100 U/ml penicillin, 100 μg/ml streptomycin, 1% glutamine, 1% nonessential amino acids, and geneticin at a final concentration of 1 μg/ml.

### Lentiviral vector production

The lentiviral CRISPR/Cas9 nickase-mediated BIRC5 gene editing vectors were constructed by annealing four gRNA oligonucleotide pairs and subcloning them in the BsmII site of lentiviral vector Lentiguide-puro vector (#52963, Addgene), and gRNAs were driven by human U6 promoter. Two gRNA sequences, 5’CGGGTCCCGCGATTCAAATC and 5’AGAGGTGGCGGCGGCGGCAT, were designed. CRISPRcas9 nickase was in a separate vector, LentiCas9-blast (#52962, Addgene) and driven by EF1a promoter. Lentiviral BIRC5 overexpression vector was purchased from Applied Biological Materials Inc (Richmond, Canada). Lentivirus was produced by packaging in 293FT cells, as published previously [46]. Stable cell lines were generated by transducing the SKOV3 and OVCAR3 cells with the lentiviral CRISPR/Cas9 nickase-mediated BIRC5 gene editing and lentiCas9-blast Cas9 nickase vectors and selected with 5 μg/ml puromycin or 10ug/ml blasticidin. LentiCas9-blast was used as the control vector without gRNAs.

### Surveyor mutation assay

Genomic DNA was extracted from SKOV3 ovarian cancer cells transduced with lentiviral CRISPR/Cas9 nickase BIRC5 and control vectors. PCR was performed by amplifying the mutated region. The primer pairs used to detect BIRC5 mutations were 5’TGCCTAGGCCTCTCAAAGTG and 5’AAGACTTACATGGGGTCGTCA in surveyor mutation assay. PCR product was denatured and reannealed with the PCR program: 95°C denatured for 5 min, ramped down to 85°C at -2°C/s and then ramped down to 25°C at -0.1°C/s; held at 4°C. Afterwards, 10 units of T7 endonuclease I was added and incubated at 37°C for 30 min, and the reaction was stopped by adding 2 μl of 0.25M EDTA and then visualized on a 1.2% agarose gel.

### MTT assay

SKOV3 or OVCAR3 cells (8000/well) transduced with lentiviral CRISPR/Cas9 nickase for BIRC5 editing and control vectors were plated into 96-well plates and cultured at different time points (24,48 and 72h). Thereafter, 10 μl of MTT reagent was added to each well and incubated for ∼4 h and then terminated by adding 100 μl detergent reagent to incubate at 22°C in the dark for 2 h. Cell proliferation was assessed by measuring the absorbance at 570 nm wavelength.

### Cell clonogenic survival assay

400 BIRC5 knockout and control SKOV3 and OVCAR3 cells or wild type cells treated with YM155 or vehicle were seeded on 6-well plates and cultured for 2 weeks and then fixed with 70% ethanol and stained with crystal blue. Colonies were counted for statistical analysis in triplicate.

### Cell migration assay

The cell migration assay was performed using a modified transwell chamber (BD Falcon™, San Jose, CA). These chambers were inserted into 24-well cell culture plates. SKOV3 or OVCAR3 cells transduced with lentiviral BIRC5 Cas9 nickase gRNAs and control vectors (3 × 10^4^) in 300 μl serum-free DMEM were added to the upper chamber. DMEM containing 10% FBS (serving as the chemoattractant) was added into the lower chamber of each well and incubated for 24 h. The medium and nonmigrated cells in the upper chamber were removed, while the migrated cells on the lower side of the membranes were fixed with methanol and stained with crystal violet. Pictures were taken at 10X magnification, and cells from at least three different fields were counted.

### Cell invasion assay

SKOV3 and OVCAR3 (5 × 10^5^) cells transduced with lentiviral BIRC5 gRNA and control vectors were seeded in serum-free DMEM onto inserts precoated with Matrigel (BD BioCoat™ using 24-well Tumor Invasion System (BD BioSciences, San Jose, CA). DMEM containing 10% FBS was added to the bottom chamber of the invasion system as the chemoattractant. The transwell inserts were stained for 5 min with hematoxylin and eosin following methanol fixation for 20 min following overnight incubation. Pictures were taken at 10X magnification. Invaded cells were counted in at least three different fields.

### Immunofluorescent staining

To detect survivin gene expression, ovarian serous carcinoma sections were antigen-retrieved and incubated with blocking buffer (5% normal goat serum, 3% bovine serum albumin, and 0.1% Triton-X100 in PBS) for 1 h. The slides were incubated overnight with primary antibodies to survivin (1:200 dilution, Cell Signaling, Danvers, MA). After rinsing three times for 5 min with PBST, Alexa 488- or 594- conjugated goat anti-rabbit (Invitrogen, Carlsbad, CA) antibodies were added for 1 h at room temperature. Cell nuclei were counterstained with DAPI (Vector Laboratories, Inc.; Burlingame, CA). Images were captured on a Zeiss LSM700 laser scanning confocal microscope.

### Tissue microarray and immunohistochemistry

Tissue microarray (TMA) including 10 cases of high grade serous ovarian cancer tumors and 10 cases of normal fallopian tubes was prepared at Northwestern University Pathological Core Facility. TMA were sectioned at 4 μm in thickness. TMA slides were deparaffinized in xylene and rehydrated in a graded series of ethanol. After antigen retrieval, all immunohistochemical staining was performed on a Ventana Nexus automated system. In brief, endogenous peroxidase activity was blocked with 3 % hydrogen peroxide. After blocking in 1.5 % normal goat serum for 30 min at room temperature, slides were then incubated overnight at 4 °C with rabbit polyclonal anti-survivin (1:250) in a humid chamber. Staining was detected with I-View 3, 3′-diaminobenzidine (DAB) detection system. Semi-quantitative immunointensity was scored in carcinoma tumor cells and normal fallopian epithelial cells as 0 (negative), 1 (weak), 2 (moderate) and 3 (strong) and percentage was showed as %.

### Cell apoptosis

Stable SKOV3 and OVCAR3 cancer cell lines established with lentiviral BRIC5 gRNA and control vectors were treated with the chemotherapy drug paclitaxel at different doses (0, 20, 40 nM) for 24 h. Apoptosis was measured using a caspase3/7 activity assay kit (Promega, Madison, WI). Cell apoptosis was also detected in both SKOV3 and OVCAR3 cells transduced with lentiviral CRISPR/Cas9 nickase-mediated BIRC5 gRNAs and control vectors by using Western blot by detecting cleaved PARP and active caspase 3.

### Western blot

Ovarian cancer cells were collected in RIPA buffer (Thermo Scientific; Rockford, IL) containing 1% Halt Proteinase Inhibitor Cocktail (Thermo Scientific; Rockford, IL). An equal amount of protein (40 μg/lane) was loaded onto 10% SDS-PAGE gels and transferred onto nitrocellulose membranes. The membranes were blocked with 5% nonfat milk for 1 h and incubated with primary antibodies against PDCD4 (Cell Signaling), GAPDH (Sigma; St. Louis, MO), vimentin, E-cadherin, or snail2 (Cell Signaling), cytokeratin-7.

### Statistical analysis

Significant differences were determined from at least two independent experiments performed in triplicate and presented as means ± SD by using Student’s *t-*test. *P* < 0.05 was considered significant.

## SUPPLEMENTARY MATERIALS FIGURE


