# Supplementary material for: Lentiviral CRISPR/Cas9 nickase vector mediated BIRC5 editing inhibits epithelial to mesenchymal transition in ovarian cancer cells
Source: Oncotarget. 2017 Oct 17;8(55):94666–80. doi: 10.18632/oncotarget.21863 (PMC5706903; doi:10.18632/oncotarget.21863)
Supplement: Supplementary file 1 [file oncotarget-08-94666-s001.pdf]

Lentiviral CRISPR/Cas9 nickase vector mediated BIRC5 editing inhibits epithelial to mesenchymal transition in ovarian cancer cells

SUPPLEMENTARY MATERIALS

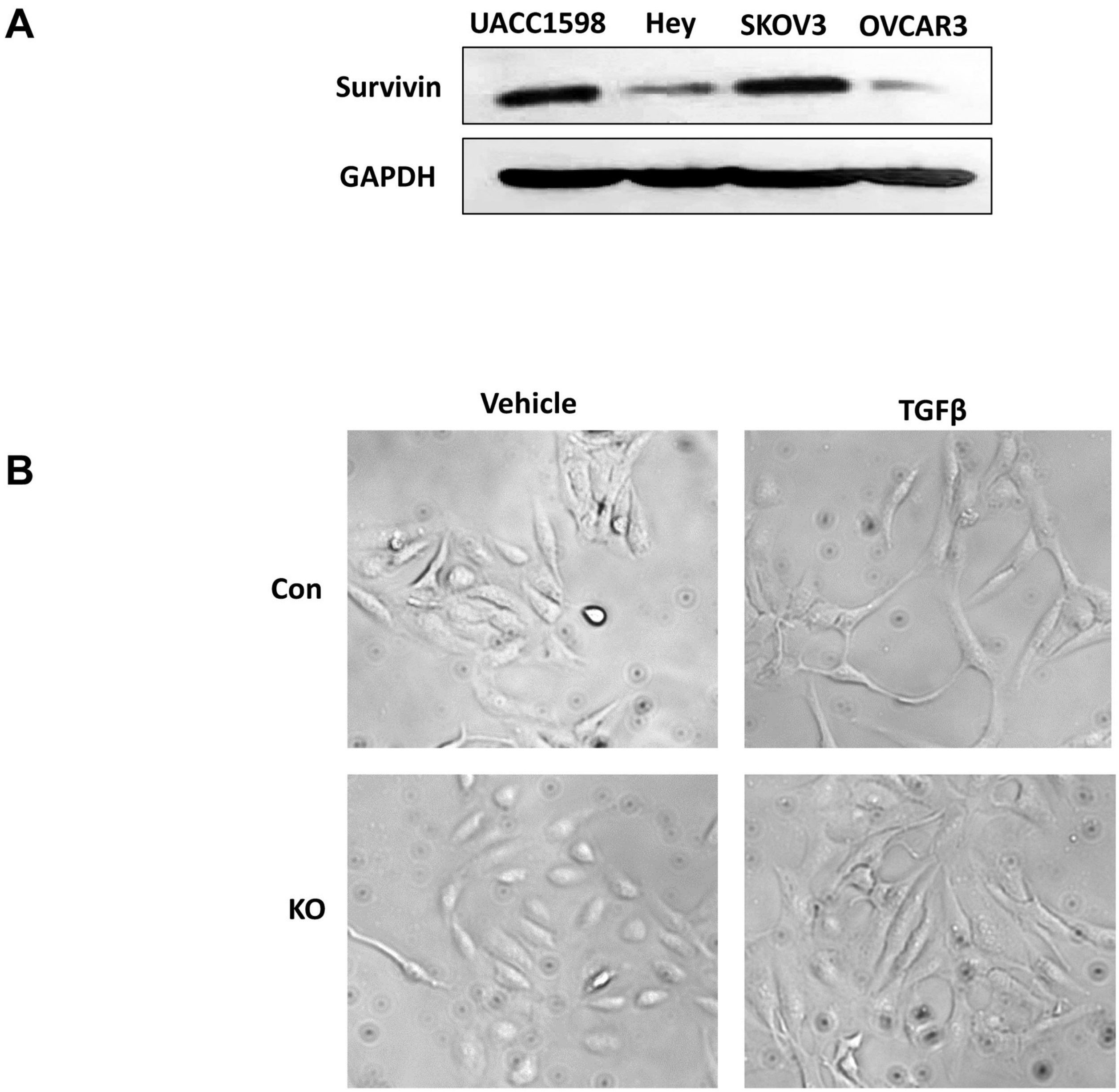

Supplementary Figure 1: BIRC5 expression in ovarian cancer cell lines. **(A)** The endogenous BIRC5 expression in ovarian cancer cell lines was examined by Western blot. **(B)** Ovarian cancer cell SKOV3 morphologies in BIRC5 knockout and control cells. SKOV3 BIRC5 knockout and control cells were treated with 10ng/ml of TGFβ for 48h. Cell morphology was imaged under microscopy.
